# Establishment of a Generalizable Industrial Crop Model for Microwave Extraction of Unsaturated Fatty Acids

**DOI:** 10.1155/2024/5979156

**Published:** 2024-09-26

**Authors:** Junyi Chen, Didi Lu, Shiqiang Chen, Song Liu, Yaqiu Zhang, Conghong Zhan

**Affiliations:** ^1^ College of Intelligent Systems Science and Engineering Hubei Minzu University, Enshi 445600, China; ^2^ College of Biological and Agricultural Engineering Jilin University, Changchun 130012, China; ^3^ College of Chemistry Jilin University, Changchun 130012, China

**Keywords:** alpha-linolenic acid, linoleic, microwave extraction, oleic, response surface, unsaturated fatty acid

## Abstract

To explore the relationship between unsaturated fatty acid (UFA) content and parameters for microwave extraction, multimaterial and multiparameter testing was conducted in which five kinds of materials with different UFA contents (potato, wheat, corn, soybean, and peanut) were selected for the experiment. Four factors, namely, extraction temperature (*X*_1_), extraction time (*X*_2_), proportional volume of acetone (*X*_3_), and liquid-to-solid ratio (*X*_4_), were screened for their significant effects by using Prob > |*T*| values from the Plackett–Burman experiment. A microwave extraction orthogonal experiment with four factors and five levels was designed separately using Design-Expert 8.05 software and them concentrated. Microwave-accelerated methyl esterification was then performed, and the UFA content was determined via gas chromatography (flame ionization detector). The optimal extraction conditions (*X*_1_, *X*_2_, *X*_3_, *X*_4_) and the optimal UFA content of potato were 80.68°C, 10.74 min, 0.80, 3.25 mL × g^−1^, 1.08%; wheat: 80.81°C, 10.54 min, 0.80, 20.91 mL × g^−1^, 2.26%; corn: 81.18°C, 9.93 min, 0.80, 50.94 mL × g^−1^, 6.89%; soybean: 82.07°C, 9.07 min, 0.80, 93.87 mL × g^−1^, 15.81%; and peanut: 83.12°C, 8.11 min, 0.80, 168.70 mL × g^−1^, 33.07%. Then, the optimization results for the five kinds of materials were synthesized by Origin 8.0 software, the fitting degree of the cubic model with the four extraction factors was the highest, the determination coefficients were 0.9984, 0.9991, 0.8953, and 0.9989, the residual sums of squares were 0.2888, 0.1587, 0.8265, and 0.1864, and the correlation coefficients are ideal. The stability and accuracy of the model were verified by the orthogonal experiment of UFA extraction from rice, and the correlation coefficient between the predicted value and the actual value of the orthogonal experiment was 0.9998. This study systematically collates the optimal parameters for microwave extraction of UFA content in different crops from the perspective of multimaterial and multiparameter, which can provide a large amount of detailed basic data for microwave extraction technology.

## 1. Introduction

Plants contain large quantities of unsaturated fatty acids (UFAs), mainly linoleic acid, oleic acid, and *α*-linolenic acid [1–3]. These are very important dietary fatty acids with important physiological functions. The quantity of UFAs directly determines the nutritional quality of plant material [4]. Additionally, extraction is a very important tool in the study of unsaturated fat processes in materials [5, 6]. Microwave extraction is widely used because of its advantages, including (1) time and energy efficiency; (2) strong selectivity; (3) low consumption of reagents and environmental friendliness; (4) high yield and recovery rate; and (5) ease of process control [7, 8].

Microwave extraction is a process in which electromagnetic waves penetrate the extraction medium and reach the interior of the material, causing the temperature inside the cells to rapidly rise, leading to cell disruption and the free flow of active ingredients. So in microwave extraction, many parameters, such as extraction temperature and extraction time, affect the extraction results [9, 10]. Therefore, it is necessary to optimize these parameters to maximize material extraction. Many studies on parameter optimization based on various methods and software have been conducted [11, 12], including the extraction of PAHs from marine sediments [13, 14], the extraction of flavonoids from sweet potato [15], the extraction of PAHs from fish [16], and the extraction of taxanes from *Taxus cuspidata* needles [17]. Previous research on the microwave extraction of UFAs has been scattered. Most of the studies have focused on a single extraction condition (such as extraction temperature or extraction time) or a single material but have not investigated the extraction process as a whole [9, 10, 18].

On the basis of previous studies, this study systematically collates the optimal parameters for microwave extraction of unsaturated fatty acid (UFA) content in different crops from the perspective of multimaterial and multiparameter, not only several factors that have a significant influence on the extraction results but also the differences caused by various extraction conditions for different materials, and a relatively complete and applicable general formula for the microwave extraction of UFAs is established. This study is a comprehensive study on the unified standardization of microwave extraction technology for UFAs, and the use of a large amount of basic data as theoretical support is also a highlight of this article.

This study aims to explore the relationship between UFA content and parameters for microwave extraction. Five kinds of materials with different UFA contents (potato, wheat, corn, soybean, and peanut) were selected for microwave extraction orthogonal experiment combined with the central composite design (CCD) method and response surface methodology (RSM). The optimal extraction process for each material was determined through the establishment of a response surface. Then, the data from all materials were integrated to establish a new generalized model, which revealed the relationship between UFA content and extraction parameters. The relationship can be directly applied to microwave extraction of UFAs from other materials to select the corresponding process parameters.

## 2. Materials and Methods

### 2.1. Materials

#### 2.1.1. Materials

In order to enhance the universality and applicability of the laws and models obtained in this study, we selected five experiment materials: potato tuber, wheat caryopsis, corn grain, soybean seed, and peanut seed (hereinafter referred to as potatoes, wheat, corn, soybeans, and peanuts) with oil contents of 0.1%-1% [19, 20], 2%-3% [21], 4%–8% [22], 17%–23% [23–25], and 40%–57% [26], respectively, and the approximate UFA contents of the oils were 66% [20], 80% [21], 70%–84% [22], 60%–80% [23, 25], and 70%–85% [26].

Potatoes (Zhong Shu No. 5), wheat (Chang Chun 413), corn (Zheng Dan 958), soybeans (Fu Dou No. 9), and peanuts (Shan Hua No. 9) were dried with the secondary drying method until the water content was reduced below the safe moisture level for each material; the moisture content is about 15% [27]. The samples were then pulverized by a Tube Mill control system (IKA, Staufen, Germany) and finally passed through a standard 0.150-mm sieve.

#### 2.1.2. Reagents

Linoleic acid (97%) and *α*-linolenic acid (97%) were purchased from Aladdin Reagent (Shanghai) Ltd. (Shanghai, China). Analytically pure oleic acid (97%), *n*-hexane, acetone, concentrated sulfuric acid, and methanol were obtained from Sinopharm Chemical Reagent Co., Ltd. (Shanghai, China). Nitrogen (99.99%) was purchased from Changchun Gas Manufacturing Co., Ltd. (Changchun, China).

#### 2.1.3. Instruments

The following instruments were used: a TANK microwave digestion system (Jinan Hanon Instruments Co., Ltd., Jinan, China), an Agilent 6890N gas chromatograph (Agilent Technologies, Shanghai, China), a JDKY-I thin-layer drying bench (Changchun Jida Scientific Instruments Equipment Co., LTD., Changchun Jilin 2, China), an OS-200 orbital shaker and MD200-1 nitrogen evaporator sample concentrator (Hangzhou Allsheng Instruments Co., Ltd., Hangzhou, China), a TG16K tabletop high-speed centrifuge (Changsha Dongwang Experimental Instrument Co., Ltd., Changsha, China), an SC-15 digital control circulator water bath (Ningbo Scientz Biotechnology Co., Ltd., Ningbo, China), a CHD100 dicer (Shandong Yinying Cooking Machinery Co., Ltd., Shandong, China), and a Tube Mill control system (IKA).

### 2.2. Orthogonal Experimental Design

#### 2.2.1. Plackett‒Burman Experiment

Considering the quality deterioration of the samples as well as the cost of time, in this study, potato was used as the sample for microwave extraction experiments in conjunction with the Plackett‒Burman experiment (Design-Expert software version 8.0.5, Stat-Ease Inc., Minneapolis, MN, USA) [28], and based on the value of Prob > |*T*|, four factors with significant effects were screened from six factors (microwave power, sample weight, extraction temperature, extraction time, proportional volume of acetone, and liquid-to-solid ratio), namely, extraction temperature, extraction time, proportional volume of acetone (acetone:hexane), and liquid-to-solid ratio (extraction solvent volume in millilitres:material mass in grams) (Table 1).

#### 2.2.2. CCD

Based on the Plackett‒Burman experiment, four experiment factors were selected, and the corresponding value interval of each factor was determined. Considering the four-factor, five-level experimental conditions selected in this study, and the interactions between factors, the CCD method [29] was used to design an orthogonal experiment (Table 2), and a regression model was generated. Regression equations were obtained, and the optimal extraction conditions for the extraction of UFAs from each material were determined.

### 2.3. Determination of UFAs

**Figure 1:**
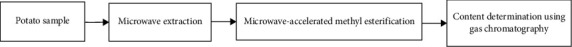
Flowchart for the detection of unsaturated fatty acids in materials (with potato taken as an example).

Taking potatoes as an example, the determination procedure is shown in Figure 1 [30].

#### 2.3.1. Extraction of UFAs

First, 25 mL of extraction solvent was added to the microwave extraction tank, and then, the potato sample was added. The UFAs were extracted by microwave extraction. The mixture ratio of *n*-hexane and acetone in the extraction solvent (proportional volume of acetone), the ratio of extraction solvent to material (liquid-to-solid ratio), the extraction temperature, and the extraction time were selected according to the parameters in Table 2. After extraction, the precipitate was filtered off, and the supernatant was retained by centrifugation and then concentrated to 2 mL with a nitrogen blower [9].

#### 2.3.2. Methyl Esterification of UFAs

The concentrated 2 mL sample was moved into the microwave tank, and then, 15 mL methyl esterification reagent (the volume ratio of concentrated sulfuric acid to methanol was 1:20; incubation was performed at 80°C for 20 min) was added to the tank for microwave methyl esterification. After methyl esterification was completed, 5 mL of *n*-hexane was added to the tank, the upper solution layer was removed by a liquid-transfer pipette, and the supernatant was obtained after centrifugation [31].

#### 2.3.3. Determination of UFAs by Gas Chromatography

The content of UFAs in the solution after centrifugation was determined by gas chromatography with an FID. Report all the features of the column: chromatographic column: HP-5; injection port temperature: 200°C; shunt ratio: 20:1; detector temperature: 290°C; injection volume: 1 *μ*L; column temperature: initial temperature: 150°C and maintained for 1 min, 20°C/min to 200°C and maintained for 1 min, 3°C/min to 230°C and maintained for 1 min, 20°C/min to 240°C and maintained for 1 min; and carrier gas: high-purity nitrogen, flow rate: 1.2 mL/min. Then, the content of each component was calculated using the gas chromatography area normalization method [32].

### 2.4. Data Processing and Optimization

The measured UFA contents were analyzed by ANOVA with Design-Expert 8.05 software [33], and the response surface and regression model were established. The UFA extraction process of potato was optimized by the response surface method, and the optimal extraction parameters were obtained [10].

### 2.5. Data Summarization and Model Establishment

For the wheat, corn, soybean, and peanut samples, the steps described in Sections 2.2–2.4 were repeated according to the process used for the potato sample. The optimal extraction process parameters for the five kinds of materials were synthesized, nonlinear fitting was performed by Origin 8.0 software (OriginLab Corporation, Northampton, MA, USA), the best fitting method was selected, and the relationship between UFA content and the optimal extraction process parameters was established [34].

## 3. Data Processing and Analysis

### 3.1. Data Processing and Analysis of the Orthogonal Experiment

#### 3.1.1. Data Results

The orthogonal experiment data for the five materials are shown in Table 3. ANOVA was performed using Design-Expert 8.05 software (Stat-Ease, Inc.). The results of the significance experiment of the regression model are shown in Table 4.

As seen from Table 4, when the *p* value is ≤ 0.05, the model of the corresponding item is significant, on the contrary, it is not significant, and the smaller the *p* value, the more significant the model is; when the *p* value of the lack of fit is ≥ 0.05, the experimental error can be regarded as a systematic error, and the higher the *p* value of the lack of fit, the smaller the experimental error [35, 36]. At the same time, it is considered in combination with the values of indicators such as *F* value, df, *R*-squared, and Adj *R*-squared. Therefore, the models of the five materials resulting from the orthogonal experiment are significant, and the lack of fit is not significant, indicating that the models are statistically significant and highly reliable. Three factors, namely, extraction temperature, volumetric proportion of acetone, and liquid-to-solid ratio, have significant effects on the extraction of UFAs in the five materials. However, the effects of extraction time and interactions between factors are not significant (*p* > 0.05).

#### 3.1.2. Multiple Regression Fitting

The regression equations are shown in Table 5, the correlation coefficients of the regression equations are all above 0.85. The relationship between the content of UFAs and *X*_1_ (extraction temperature), *X*_2_ (extraction time), *X*_3_ (proportional volume of acetone), and *X*_4_ (liquid-to-solid ratio) was fitted by multiple regression fitting. The results showed that all model *R*^2^ values were more than 0.85, especially the wheat *R*^2^ value, which was 0.9883, proving that the design of the experiment was scientific and reasonable.

#### 3.1.3. Analysis of Response Surface

**Figure 2:**
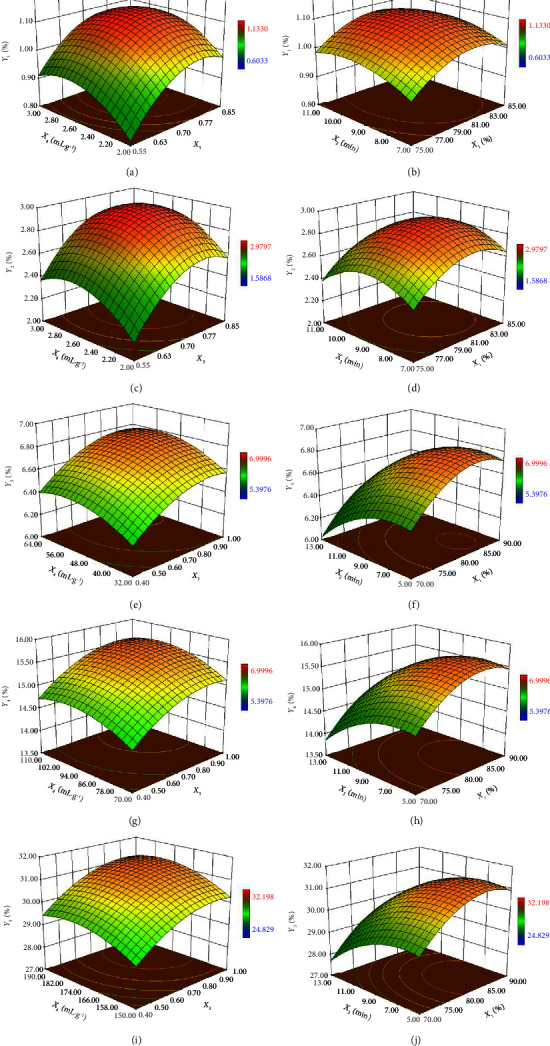
Relationship between various factors and unsaturated fatty acid content of corn.

The response surfaces are shown in Figure 2, and the following conclusions can be obtained from the response surfaces in Figure 2:

1. Extraction temperature: The extraction temperature had a significant effect on the extraction rate of UFAs from the five materials (*p* < 0.05). The extraction rate of UFAs from each material initially increased and then decreased with increasing extraction temperature (Figures 2(a), 2(c), 2(e), 2(g), and 2(i)). The reason for this result is that all five materials contain lipoxygenase, which can catalyze the oxidation of PUFAs to form ROOH, which corresponds to the process from polyunsaturated to monounsaturated to saturated [37–40].
2. Extraction time: The extraction time had no significant effect on the extraction rate of UFAs from the five materials (*p* > 0.05). The extraction rate of UFAs from each material slowly increased initially and then stabilized with increasing extraction time (Figures 2(a), 2(c), 2(e), 2(g), and 2(i)). This is related to the degree of decomposition of the material by microwave radiation and the amount of oil diffusing to the surface of the material [10].
3. Proportional volume of acetone: The proportional volume of acetone had a significant effect on the extraction rate of UFAs from the five materials (*p* < 0.05). The extraction rate of UFAs in each material increased initially and then decreased with increasing proportional volume of acetone (Figures 2(b), 2(d), 2(f), 2(h), and 2(j)). The reason for this phenomenon is that the dielectric constants of acetone and *n*-hexane are different, resulting in different rates of solute heating, which affects the oxidative decomposition of lipids [41, 42].
4. Liquid-to-solid ratio: The liquid-to-solid ratio had a significant effect on the extraction rate of UFAs from the five materials (*p* < 0.05). The extraction rate of UFAs from each material increased initially and then decreased with increasing liquid-to-solid ratio (Figures 2(b), 2(d), 2(f), 2(h), and 2(j)). This occurred because the extraction rate is related to the diffusion of the oils from the material into the extraction solvent, involving the difference between the concentrations of oil and osmotic pressure [43].

### 3.2. Establishment of the Model Between the Extraction Process and the Content of UFAs

#### 3.2.1. Optimal Extraction Scheme for Each Material

The optimization scheme for the UFA extraction process was determined by the optimization-numerical function, and the optimal values for the microwave extraction of UFA under this scheme were determined, the synthesis of the optimal extraction process actual values and predicted values for the five kinds of materials is shown in Table 6.

#### 3.2.2. Establishment of Extraction Model

By using a custom function in Origin 8.0 software, the optimal process parameters for the microwave extraction of UFAs in the five kinds of materials were analyzed by nonlinear fitting. The fitting accuracies of six exponential, asymptotic, and growth/anticurve functions with respect to that of single extraction factors were systematically compared, including the RSS and *R*^2^, and the universality was investigated. The larger *R*^2^ is, the smaller RSS is, and the better the fitting formula matches the data of each extraction factor. The extraction factors correspond to *X*, and the content of UFAs is *Y*. The results are shown in Table 7.

**Figure 3:**
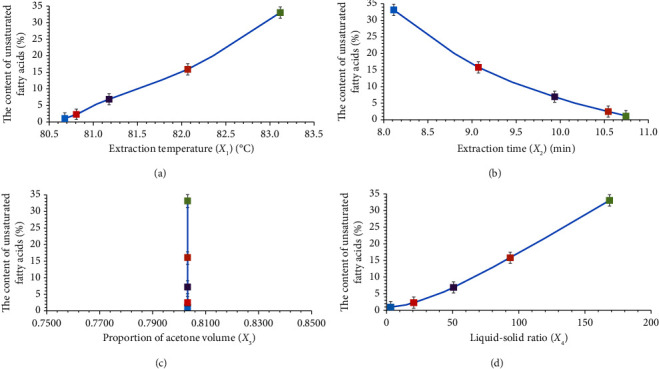
Trend diagrams of the values of each factor when the yields of unsaturated fatty acids for the five kinds of materials are the highest.

The fitting degree of the cubic model to the data for the four extraction factors (*X*_1_, *X*_2_, *X*_3_, *X*_4_) is the highest; the determination coefficients are 0.9984, 0.9991, 0.8953, and 0.9989, and the residual sums of squares (RSSs) are 0.2888, 0.1587, 0.8265, and 0.1864. The correlation between UFA content and the optimal value of each factor obtained by using the cubic model is quite ideal. Furthermore, the optimal value of each factor is consistently related to the UFA content in each material. Therefore, we chose the cubic model to fit the relationship between the UFA content and the extraction factors, then determined the formulas (Table 8), and drew the corresponding diagram (Figure 3).

#### 3.2.3. Analysis and Discussion of the Model

The results showed that during microwave extraction, a higher UFA content (*Y*) was associated with a higher optimal extraction temperature (*X*_1_), a shorter optimal extraction time (*X*_2_), and a higher optimal liquid-to-solid ratio (*X*_4_); however, the optimal proportional volume of acetone (*X*_3_) did not vary.

1. Extraction temperature: The higher the content of UFAs is, the higher the optimal extraction temperature is. Because the fatty acid composition of each material is different, oil oxidation is related to the number, position, and geometry of the double bonds of its constituent fatty acids. The greater the number of double bonds is, the faster the oxidation rate is. The ratio of the oxidation rates of *α*-linolenic acid, linoleic acid, and oleic acid is approximately 20:10:1 [44, 45]. Therefore, the order of optimal extraction temperatures from high to low was peanut > soybean > corn > wheat > potato.
2. Extraction time: The higher the content of UFAs is, the shorter the optimal extraction time is, which is the opposite of the trend observed for the optimal extraction temperature. Therefore, the order for the optimal extraction time from short to long was peanut < soybean < corn < wheat < potato.
3. Proportional volume of acetone: The optimal acetone ratios of materials with different contents of UFAs are basically the same. This may be because the optimal proportional volume of acetone is related only to the extraction temperature and the extraction efficiency but has little to do with the content of UFAs. For all five materials, the highest extraction rate of UFAs was achieved when the optimal proportional volume of acetone was approximately 0.8.
4. Liquid-to-solid ratio: The higher the content of UFAs is, the higher the liquid-to-solid ratio is. Therefore, the order for the optimal liquid-to-solid ratio from high to low was peanut > soybean > corn > wheat > potato.

#### 3.2.4. Application and Promotion of the Model

These results suggest that the changes in the various factors follow a predictable pattern, and this pattern is related to the UFA content of the material. For the microwave extraction of UFAs from other materials, the optimal extraction conditions can be obtained by substituting the approximate UFA content of the material into the four cubic models, and the maximum extraction of UFAs can be achieved using the derived optimum extraction scheme.

### 3.3. Verification of Experiment Feasibility

#### 3.3.1. Model Validation Experiment

Rice is one of the three major staple grains in China. A large number of studies have proved that the UFA content of rice is around 5% to 7%. In this study, the stability and accuracy of the model were verified by the orthogonal experiment of UFA extraction from rice (Table 9). The orthogonal experiment data are shown in Table 10, and the optimal extraction process and optimal extraction results were solved by ANOVA and fitting. At the same time, we brought the optimal extraction results into the model in Table 8 and solved the predicted value of the optimal extraction scheme. The correlation coefficient (CORREL function) between the predicted value and the actual value of the orthogonal experiment was 0.9998 (Table 11), which proved that the model effect was effective.

#### 3.3.2. Comparative Experiment

Taking potato and peanut as samples, the microwave extraction and Soxhlet extraction (national standard) methods were compared; the factors of the microwave method were the optimal ones obtained above, whereas the factors of the Soxhlet extraction method were those used in the national standard. The results are shown in Table 12. The extraction results show that compared with the Soxhlet extraction method, the microwave extraction method not only uses a smaller amount of reagent but also shortens the extraction time, and the extraction results are ideal.

#### 3.3.3. Recovery Rate Experiment

The recovery experiment results are shown in Table 13. Taking peanuts as an example, the standard product recovery rate has a good value of 96.54%–100.9% (see Table 14).

## 4. Conclusion

This study established a generalizable industrial crop model for microwave extraction of UFAs. Firstly, in this study, the Plackett‒Burman screening experiment was utilized to screen four factors with significant effects from six factors by the value of Prob > |*T*|, which were extraction temperature *X*_1_, extraction time *X*_2_, proportional volume of acetone *X*_3_, and liquid-to-solid ratio *X*_4_. Design of microwave extraction orthogonal experiment using CCD method, the fitting degree of the cubic model with the data for four extraction factors (*X*_1_, *X*_2_, *X*_3_, *X*_4_) is the highest; the determination coefficients are 0.9984, 0.9991, 0.8953, and 0.9989, and the RSSs are 0.2888, 0.1587, 0.8265, and 0.1864. The correlation between the UFA content and the optimal value of each factor obtained by using the cubic model is quite ideal, and the regression equations are as follows: *Y*=−945050.5889+34760.3618*X*_1_ − 426.2936*X*_1_^2^+1.7431*X*_1_^3^; *Y*=1065.0620 − 284.5030*X*_2_+25.9215*X*_2_^2^ − 0.8060*X*_2_^3^; *Y*=(5.8753 − 21.932*X*_3_+27.29*X*_3_^2^ − 11.319*X*_3_^3^) × 10^9^; *Y*=0.8583+0.0389*X*_4_+0.0018*X*_4_^2^ − 5.0746 × 10^−4^*X*_4_^3^, where the correlation coefficients are ideal. The higher the content of UFAs is, the higher the optimal extraction temperature is. The higher the content of UFAs is, the shorter the optimal extraction time is. The optimal acetone ratios of materials with different contents of UFAs are basically the same. The higher the content of UFAs is, the higher the liquid-to-solid ratio is. Finally, the correlation coefficient between the actual values of the rice experiment and the predicted values of the cubic model was 0.9998, which fully verified the accuracy as well as the generalizability of the model.

Most previous studies focused on a single extraction condition or a single material, but this study systematically collates the optimal parameters for microwave extraction of UFA content in different crops from the perspective of multimaterial and multiparameter. This model not only considers many factors that have a significant influence on the extraction results but also considers the difference caused by extraction conditions between different materials. A model of the unsaturated fat microwave extraction process has been established, which can not only help perfect the theory of unsaturated fat extraction but also provide guidance for microwave extraction technology.

However, as we emphasized in the main text, this generalized model was established on the experimental data of five materials. If the model can be further improved through more extraction experiments with different materials in the future, its generality and accuracy will definitely be better.

## Figures and Tables

**Table 1:** Results of the Plackett‒Burman test screening.

**Factor**	**Unit**	**Coded level**		**T-test**	**Prob > |T|**	**Ranking**
		**−1**	**+1**			
Microwave power	W	500	800	1.1246	0.3376	5
Sample weight	g	2	6	0.9376	0.4731	6
Extraction temperature	°C	70	90	1.8268	0.0964	1
Extraction time	min	5	13	1.1990	0.3328	4
Volumetric proportion of acetone	—	0.4	1.0	1.5409	0.1132	2
Liquid-to-solid ratio	mL × g^−1^	1.5	3.5	1.5033	0.1462	3

**Table 2:** Coded levels of factors related to extraction conditions for the five materials.

**Level**	**Factors**							
	**Extraction temperature** **X**_1_ **(°C)**	**Extraction time** **X**_2_ **(min)**	**Proportional volume of acetone** **X**_3_	**Liquid**-to-**solid ratioX**_4_**(mL·g**^−1^**)**				
				**Potato**	**Wheat**	**Corn**	**Soybean**	**Peanut**
−2	70	5	0.40	1.5	14	32	70	150
−1	75	7	0.55	2.0	17	40	80	160
0	80	9	0.70	2.5	20	48	90	170
1	85	11	0.85	3.0	23	56	100	180
2	90	13	1.00	3.5	26	64	110	190

**Table 3:** Experimental design and response values for the five materials.

**Test number**	**Level**				**Y (%)**				
	**X**_1_	**X**_2_	**X**_3_	**X**_4_	**Potato**	**Wheat**	**Corn**	**Soybean**	**Peanut**
1	−1	−1	−1	−1	0.71	1.68	5.82	13.39	26.79
2	1	−1	−1	−1	0.78	1.93	6.46	14.86	29.72
3	−1	1	−1	−1	0.68	1.66	5.40	12.41	24.83
4	1	1	−1	−1	0.75	1.92	5.81	13.36	26.71
5	−1	−1	1	−1	0.89	2.15	6.60	15.18	30.36
6	1	−1	1	−1	0.96	2.41	6.40	14.71	29.43
7	−1	1	1	−1	0.83	2.09	5.72	13.15	26.30
8	1	1	1	−1	0.90	2.33	6.28	14.44	28.87
9	−1	−1	−1	1	0.75	1.87	6.09	14.02	28.03
10	1	−1	−1	1	0.87	2.13	6.38	14.67	29.33
11	−1	1	−1	1	0.73	1.82	5.72	13.15	26.29
12	1	1	−1	1	0.82	2.15	6.14	14.13	28.26
13	−1	−1	1	1	0.98	2.39	6.55	15.06	30.12
14	1	−1	1	1	1.04	2.51	7.00	16.10	32.20
15	−1	1	1	1	0.95	2.36	6.11	14.05	28.11
16	1	1	1	1	1.00	2.48	6.29	14.46	28.92
17	−2	0	0	0	0.82	1.94	5.53	12.72	25.44
18	2	0	0	0	1.01	2.39	6.44	14.80	29.60
19	0	−2	0	0	1.00	2.19	6.18	14.21	28.42
20	0	2	0	0	0.82	2.13	6.18	14.21	28.42
21	0	0	−2	0	0.64	1.59	5.98	13.76	27.53
22	0	0	2	0	0.99	2.34	6.51	14.96	29.92
23	0	0	0	−2	0.76	1.86	6.25	14.37	28.73
24	0	0	0	2	1.04	2.45	6.38	14.67	29.34
25	0	0	0	0	1.03	2.98	6.86	15.78	31.56
26	0	0	0	0	0.97	2.81	6.71	15.43	30.87
27	0	0	0	0	0.99	2.94	6.74	15.51	31.02
28	0	0	0	0	0.94	2.86	6.65	15.29	30.58
29	0	0	0	0	0.99	2.89	6.68	15.36	30.71
30	0	0	0	0	0.93	2.81	6.74	15.50	31.01

*Note:X*_1_: extraction temperature, °C; *X*_2_: extraction time, min; *X*_3_: proportional volume of acetone; *X*_4_: liquid-to-solid ratio, mL × g^−1^; Y: unsaturated fatty acid content, %.

**Table 4:** Significance of the test results of the regression models for the five materials.

**Source**	**Materials**	**Potato**	**Wheat**	**Corn**	**Soybean**	**Peanut**
*p* value Prob > *F*	Model	0.0006	<0.0001	<0.0001	0.0003	0.0003
	*X*_1_	0.0005	<0.0001	<0.0001	0.0004	0.0004
	*X*_2_	0.1122	0.0647	0.1243	0.0916	0.0874
	*X*_3_	0.0012	<0.0001	<0.0001	0.0008	0.0013
	*X*_4_	0.0057	0.0007	<0.0001	0.0027	0.0034
	*X*_1_ *X*_2_	0.3405	0.4439	0.7982	0.2277	0.2277
	*X*_1_ *X*_3_	0.3784	0.3863	0.1508	0.1607	0.2607
	*X*_1_ *X*_4_	0.6385	0.6015	0.4187	0.4362	0.4362
	*X*_2_ *X*_3_	0.2929	0.3781	0.3868	0.2789	0.2789
	*X*_2_ *X*_4_	0.3091	0.3393	0.3200	0.3985	0.3985
	*X*_3_ *X*_4_	0.1954	0.1803	0.1257	0.1914	0.1914
	*X*_1_^2^	0.0004	<0.0001	<0.0001	0.0002	0.0002
	*X*_2_^2^	0.0649	0.0923	<0.0001	0.0028	0.0033
	*X*_3_^2^	0.0112	<0.0001	<0.0001	0.0067	0.0084
	*X*_4_^2^	0.0125	<0.0001	<0.0001	0.0161	0.0201

Lack of fit		0.0934	0.4088	0.7747	0.1785	0.2747

*F* value		43.96	90.42	6.93	20.15	40.52

df		29	29	29	29	29

Std. dev.		0.01	0.06	0.20	0.21	0.11

C.V. (%)		3.41	2.65	3.25	5.37	3.62

*R*-squared		0.9762	0.9883	0.8660	0.9287	0.9016

Adj *R*-squared		0.9540	0.9774	0.7410	0.8806	0.8755

Pred *R*-squared		0.8896	0.9557	0.5539	0.8202	0.8065

*Note:X*_1_: extraction temperature, °C; *X*_2_: extraction time, min; *X*_3_: proportional volume of acetone; *X*_4_: liquid-to-solid ratio, mL × g^−1^.

**Table 5:** Regression equations and correlation coefficients between the content of unsaturated fatty acids in the five kinds of materials and various factors.

**Material**	**Regression model**	**Correlation coefficient**
Potato	*Y* = +0.8207 + 0.11773 ∗ *X*_1_ + 0.032533 ∗*X*_2_ + 1.24736 ∗*X*_3_ + 0.41963 ∗*X*_4_ + 0.077928 ∗*X*_3_*X*_4_ − 0.00712 ∗*X*_1_^2^ − 0.00776 ∗*X*_2_^2^ − 0.68474 ∗*X*_3_^2^ − 0.0859 ∗*X*_4_^2^	0.9240
Wheat	*Y* = +1.76 + 0.23 ∗*X*_1_ − 0.031 ∗*X*_2_ + 0.42 ∗*X*_3_ + 0.23 ∗*X*_4_ + 0.00782 ∗*X*_1_*X*_2_ − 0.046 ∗*X*_1_*X*_3_ − 0.020 ∗*X*_1_*X*_4_ − 0.017 ∗*X*_2_*X*_3_ + 0.011 ∗*X*_2_*X*_4_ − 0.00285 ∗*X*_3_*X*_4_ − 0.36 ∗*X*_1_^2^ − 0.36 ∗*X*_2_^2^ − 0.46 ∗*X*_3_^2^ − 0.36 ∗*X*_4_^2^	0.9883
Corn	*Y* = +6.73 + 0.19 ∗*X*_1_ − 0.16 ∗*X*_2_ + 0.17 ∗*X*_3_ + 0.086 ∗*X*_4_ + 0.025 ∗*X*_1_*X*_2_ − 0.048 ∗*X*_1_*X*_3_ − 0.00416 ∗*X*_1_*X*_4_ − 0.029 ∗*X*_2_*X*_3_ + 0.020 ∗*X*_2_*X*_4_ + 0.00709 ∗*X*_3_*X*_4_ − 0.19 ∗*X*_1_^2^ − 0.14 ∗*X*_2_^2^ − 0.12 ∗*X*_3_^2^ − 0.11 ∗*X*_4_^2^	0.966 0
Soybean	*Y* = +15.48 + 0.44 ∗*X*_1_ − 0.37 ∗*X*_2_ + 0.40 ∗*X*_3_ + 0.20 ∗*X*_4_ + 0.058 ∗*X*_1_*X*_2_ − 0.11 ∗*X*_1_*X*_3_ − 0.00956 ∗*X*_1_*X*_4_ − 0.067 ∗*X*_2_*X*_3_ + 0.046 ∗*X*_2_*X*_4_ + 0.016 ∗*X*_3_*X*_4_ − 0.43 ∗*X*_1_^2^ − 0.32 ∗*X*_2_^2^ − 0.28 ∗*X*_3_^2^ − 0.24 ∗*X*_4_^2^	0.8535
Peanut	*Y* = +30.96 + 0.87 ∗*X*_1_ − 0.74 ∗*X*_2_ + 0.80 ∗*X*_3_ + 0.40 ∗*X*_4_ + 0.12 ∗*X*_1_*X*_2_ − 0.22 ∗*X*_1_*X*_3_ − 0.019 ∗*X*_1_*X*_4_ − 0.13 ∗*X*_2_*X*_3_ + 0.093 ∗*X*_2_*X*_4_ + 0.033 ∗*X*_3_*X*_4_ − 0.87 ∗*X*_1_^2^ − 0.64 ∗*X*_2_^2^ − 0.56 ∗*X*_3_^2^ − 0.49 ∗*X*_4_^2^	0.8973

**Table 6:** Values of each factor corresponding to the optimal unsaturated fatty acids of the five materials.

**The optimal scheme**	**Data types**	**X**_1_ **(°C)**	**X**_2_ **(min)**	**X**_3_	**X**_4_ **(mL** **×** **g**^−1^**)**	**Y (%)**	**T test**	**P** **value**
Potato	Predicted value	80.37	10.80	0.80	3.66	1.08	0.998	<0.01
	Actual value	80.68	10.74	0.80	3.25	1.08		

Wheat	Predicted value	80.53	10.58	0.80	21.87	2.26	0.994	<0.01
	Actual value	80.81	10.54	0.80	20.91	2.26		

Corn	Predicted value	81.17	10.02	0.80	48.32	6.89	0.981	<0.01
	Actual value	81.18	9.93	0.80	50.94	6.89		

Soybean	Predicted value	81.89	9.21	0.80	96.05	15.81	0.988	<0.01
	Actual value	82.07	9.07	0.80	93.87	15.81		

Peanut	Predicted value	84.04	8.13	0.80	167.10	33.07	0.997	<0.05
	Actual value	83.12	8.11	0.80	168.70	33.07		

*Note:X*_1_: extraction temperature, °C; *X*_2_: extraction time, min; *X*_3_: proportional volume of acetone; *X*_4_: liquid-to-solid ratio, mL × g^−1^; *Y*: the optimal unsaturated fatty acid content, %.

**Table 7:** Relationship between the optimal extracted unsaturated fatty acid content and the optimal extraction process of five kinds of materials fitted by different functions.

**Fitting method**	**Fitting equation**	**Extraction temperature**		**Extraction time**		**Volumetric proportion of acetone**		**Liquid-to-solid ratio**	
		**R** ^2^	**RSS**	**R** ^2^	**RSS**	**R** ^2^	**RSS**	**R** ^2^	**RSS**
Linear	*Y*=*a*+*bX*	0.8133	97.8218	0.9394	31.7603	0.5453	91.0054	0.9811	9.9259
Quadratic	*Y*=*a*+*bX*+*cX*^2^	0.9924	2.6680	0.9979	0.7276	0.1284	204.4004	0.9964	1.2513
Cubic	*Y*=*a*+*bX*+*cX*^2^+*dX*^3^	0.9984	0.2888	0.9991	0.1587	0.8953	0.8265	0.9989	0.1864
Stirling	*Y*=*a*+b((exp *kX* − 1)/*k*)	0.9815	6.4764	0.9990	0.1842	0.0610	360.9700	0.9525	16.5878
Exp2p	*Y*=*ab*^*X*^	0.9852	7.7677	0.2540	390.8357	0.0001	1400.4500	0.7555	128.0729
Exp3p	*Y*=exp(*a*+*bX*+*cX*^2^)	0.9935	2.2827	0.5642	41.4808	0.1206	229.3660	0.9916	2.9448

**Table 8:** Equations of the relationships for the five kinds of materials corresponding to the maximum yield of unsaturated fatty acids.

**Influencing factors**	**Relationship between fatty acid yield (Y) and various factors**	**Correlation coefficient**
*X*_1_	*Y*=−945050.5889+34760.3618*X*_1_ − 426.2936*X*_1_^2^+1.7431*X*_1_^3^	0.9984
*X*_2_	*Y*=1065.0620 − 284.5030*X*_2_+25.9215*X*_2_^2^ − 0.8060*X*_2_^3^	0.9991
*X*_3_	*Y*=(5.8753 − 21.932*X*_3_+27.29*X*_3_^2^ − 11.319*X*_3_^3^) × 10^9^	0.8953
*X*_4_	*Y*=0.8583+0.0389*X*_4_+0.0018*X*_4_^2^ − 5.0746 × 10^−6^*X*_4_^3^	0.9989

*Note:X*_1_: extraction temperature, °C; *X*_2_: extraction time, min; *X*_3_: proportional volume of acetone; *X*_4_: liquid-to-solid ratio, mL × g^−1^; Y: unsaturated fatty acid content, %.

**Table 9:** Coded levels of factors related to extraction conditions for the rice.

**Level**	**Factors**			
	**Extraction temperature** **X**_1_ **(°C)**	**Extraction time** **X**_2_ **(min)**	**Proportional volume of acetone** **X**_3_	**Liquid**-to-**solid ratioX**_4_**(mL·g**^−1^**)**
−2	70	5	0.40	32
−1	75	7	0.55	40
0	80	9	0.70	48
1	85	11	0.85	56
2	90	13	1.00	64

**Table 10:** Experimental design and response values for the rice.

**Test number**	**Level**				**Yr (%)**
	**X**_1_	**X**_2_	**X**_3_	**X**_4_	
1	−1	−1	−1	−1	3.6510
2	1	−1	−1	−1	3.5515
3	−1	1	−1	−1	3.4107
4	1	1	−1	−1	3.9302
5	−1	−1	1	−1	4.4100
6	1	−1	1	−1	4.9395
7	−1	1	1	−1	4.2812
8	1	1	1	−1	4.7707
9	−1	−1	−1	1	3.8403
10	1	−1	−1	1	4.4004
11	−1	1	−1	1	3.7303
12	1	1	−1	1	4.4200
13	−1	−1	1	1	4.9094
14	1	−1	1	1	5.1403
15	−1	1	1	1	4.8507
16	1	1	1	1	5.1083
17	−2	0	0	0	3.9824
18	2	0	0	0	4.9099
19	0	−2	0	0	4.5018
20	0	2	0	0	4.3741
21	0	0	−2	0	3.2875
22	0	0	2	0	4.8147
23	0	0	0	−2	3.7913
24	0	0	0	2	5.0082
25	0	0	0	0	6.1104
26	0	0	0	0	5.7713
27	0	0	0	0	6.0382
28	0	0	0	0	5.8770
29	0	0	0	0	5.1032
30	0	0	0	0	5.7606

*Note:X*_1_: extraction temperature, °C; *X*_2_: extraction time, min; *X*_3_: proportional volume of acetone; *X*_4_: liquid-to-solid ratio, mL × g^−1^; Yr: unsaturated fatty acid content of the rice, %.

**Table 11:** Comparison between the actual value of rice extraction experiment and the predicted value of model.

**The optimal scheme**	**Actual value**	**Predicted value**	**Correlation coefficient**
Extraction temperature (°C)	83.06	82.89	0.9998
Extraction time (min)	10.02	9.99	
Proportional volume of acetone	0.78	0.80	
Liquid-to-solid ratio (mL × g^−1^)	46.12	45.60	
Optimal unsaturated fatty acid content of rice (%)	5.99	5.99	

**Table 12:** Comparison of two extraction methods.

**Extraction method**	**Material**	**Extraction solvent volume (mL)**	**Extraction temperature (°C)**	**Extraction time (min)**	**Extraction results (%)**
Soxhlet extraction	Potato	150.0	60.00	720.0	0.9744
	Peanut	150.0	60.00	720.0	31.02

Microwave extraction	Potato	20.00	80.68	10.74	1.082
	Peanut	20.00	83.12	8.110	33.06

**Table 13:** Recovery experiment results.

**Items**	**Sample value (m·** **g** ^−1^ **)**	**Added amount (mg)**	**Measured value (mg·** **g** ^−1^ **)**	**Recovery rate (%)**
Linoleic acid	0.59	0.16	0.75	100.90
Oleic acid	0.10	0.19	0.28	96.54
Alpha-linolenic acid	0.07	0.17	0.25	100.80

**Table 14:** About word abbreviations versus full word names.

**Serial number**	**Full name**	**Abbreviation**
1	Unsaturated fatty acid	UFA
2	Polycyclic aromatic hydrocarbons	PAH
3	Central composite design	CCD
4	Flame ionization detector	FID
5	Analysis of variance	ANOVA
6	*R*-square	*R* ^2^
7	Residual sum of squares	RSS

## Data Availability

The data used to support the findings of this study are included within the article.
